# Study of the Selectivity and Bioactivity of Polyphenols Using Infrared Assisted Extraction from Apricot Pomace Compared to Conventional Methods

**DOI:** 10.3390/antiox7120174

**Published:** 2018-11-27

**Authors:** Dina Cheaib, Nada El Darra, Hiba N. Rajha, Iman El-Ghazzawi, Youssef Mouneimne, Adla Jammoul, Richard G. Maroun, Nicolas Louka

**Affiliations:** 1 Faculty of Sciences, Beirut Arab University, P.O. Box 115020, Riad El Solh, Beirut 1107 2809, Lebanon; dac273@student.bau.edu.lb; 2 Faculty of Heath Sciences, Beirut Arab University, Tarik El Jedidah–Beirut, P.O. Box 115020, Riad EL Solh, Beirut 1107 2809, Lebanon; n.aldarra@bau.edu.lb (N.E.D.); i.ghazzawi@bau.edu.lb (I.E.-G.); 3 Research Unit of Agro-Food Technologies and Valorization, Analysis and Research Center, Faculty of Sciences, Saint-Joseph University of Beirut, B.P. 11-514 Riad El Solh, Beirut 1107 2050, Lebanon; hiba.rajha@usj.edu.lb (H.N.R.); nicolas.louka@usj.edu.lb (N.L.); 4 K.A.S. Central Research Science Laboratory, Department of Research Science Laboratory, American University of Beirut, P.O. Box 11-0236, Beirut, Lebanon; ym01@aub.edu.lb; 5 Food Department, Lebanese Agricultural Research Institute, Fanar, Lebanon; ajammoul@lari.gov.lb

**Keywords:** apricot pomace, polyphenols, infrared, microwaves, ultrasounds

## Abstract

The valorization of industrial food byproducts by means of environment-friendly extraction methods is becoming a major interest because of its environmental and economic values. In this study, the efficiency of many technologies, such as ultrasounds (US), microwaves (MW), and infrared (IR), was compared, in terms of polyphenol yield and bioactivity from apricot pomace. IR was the most effective method with the highest polyphenol (10 mg GAE/g DM), flavonoid (6 mg CE/g DM), and tannin (3.6 mg/L) yields. In terms of efficacy, IR was followed by MW, US, then solid-liquid (S/L) extraction. IR extract from apricot pomace exhibited the highest inhibitory activity against all the studied gram-positive strains (*Methicillin Resistant Staphylococcus aureus, Staphylococcus aureus, Methicillin-resistant Staphylococcus epidermidis,* and *Staphylococcus epidermidis*) and a one gram-negative strain (*Escherichia coli*). Moreover, IR extracts had by far the highest antiradical activity (AC) (40%) followed by MW (31%), US (28%), and then S/L (15%). High-performance liquid chromatography (HPLC) permitted the identification and quantification of rutin in all extracts; whereas catechin was detected in those of IR (3.1 μg/g DM), MW (2.1 μg/g DM), and US (1.5 μg/g DM). Epicatechin was exclusively found in IR extract (4 μg/g DM), suggesting the selectivity of IR towards this compound. Scanning electron microscopy (SEM) revealed that the IR technique induced the highest cellular and structural damage in apricot pomace, which could explain the effectiveness of this technology.

## 1. Introduction

Nowadays, the valorization of fruit byproducts has become of great interest due to their content in several bioactive molecules, such as polyphenols. Different methods have been used for the recovery of polyphenols from different matrices. Solid/liquid (S/L) extraction is the conventional process adopted in many industries for polyphenol recuperation. However, one of its main disadvantages is the massive consumption of organic solvents. The latter present a significant cost for the manufacturing. Organic solvent use in food processing is, therefore, not recommended [1]. The main research focus in recent years is the use of eco-friendly technologies that diminish organic solvent use and energy consumption. Many studies were, therefore, conducted on polyphenol recovery from different byproducts by means of emerging technologies, such as high-voltage electrical discharges (HVED), pulsed electric fields (PEF), infrared (IR), ultrasounds (US), and microwave-assisted extractions. The action mechanism of every technique is involved in the efficacy of polyphenol extraction. For example, the efficiency of ultrasounds, which are sound waves, is related to the mechanical vibrations they produce in the solid, thus having the ability to provoke structural damage and therefore enhance the extraction process of intracellular components (polyphenols, essential oils, etc.) [2]. Compared to conventional methods, US is considered as an effective operation that increases mass transfer, and reduces time and solvent consumption. For example, this technique has been found to improve the extraction yield of polyphenols ranging from 11% to 35% compared to the conventional method (solid-liquid extraction) from red grape marc [3]. Microwaves are electromagnetic radiations in the frequency between 300 MHz and 300 GHz. The microwave energy transformation to heat is caused by ionic conduction and dipole rotation, and leads to an increased solubility of the molecules of interest (such as polyphenols) in the solvent [4]. Microwave technology is a simple, time-saving method that reduces solvent consumption and energy cost. Compared to ultrasonic extraction, the microwave technique gave higher polyphenol extraction (up to four-fold) from different spices (Cinnamomum zeylanicum, Coriandrum sativum, Cuminum cyminum, and Crocus sativus) [5,6].

Infrared-assisted extraction (IRAE) technique, used as the heat source, is composed of an infrared lamp. Its highest efficiency is attributed to its high capacity of penetration [7]. Compared to other traditional extraction techniques, infrared is cheap, easy, and free of radiation, and could improve the extraction of many bioactive compounds from plants. The infrared extraction of polyphenols from rice hull showed an increase from 0.12 to 0.19 mM after 30 min and an improved the antioxidant activity from 47% to 79% compared to solid-liquid ones [8].

To the best of our knowledge, no study was yet conducted on infrared, microwave, and ultrasound-assisted extraction of polyphenols from apricot pomace. The main objective of this study was to compare the selectivity and efficiency of infrared assisted extraction on the enhancement of polyphenol recovery from apricot pomace compared to conventional techniques: Microwave, ultrasound-assisted extraction, and the solid-liquid method. The second objective was to assess their impact on biological activities (antiradical and antimicrobial activity).

## 2. Materials and Methods

### 2.1. Raw Materials

Apricot pomace (consisting of pressed skins and pulp residues) used in this study were obtained from Conserves Modernes Chtaura, Lebanon.

### 2.2. Extraction Experiments 

The extraction experiments were conducted at different times to reach the same energy consumption of 2290.90 kJ/kg for IR, MW, US, and S/L.

#### 2.2.1. Infrared-Assisted Extraction

**Figure 1:**
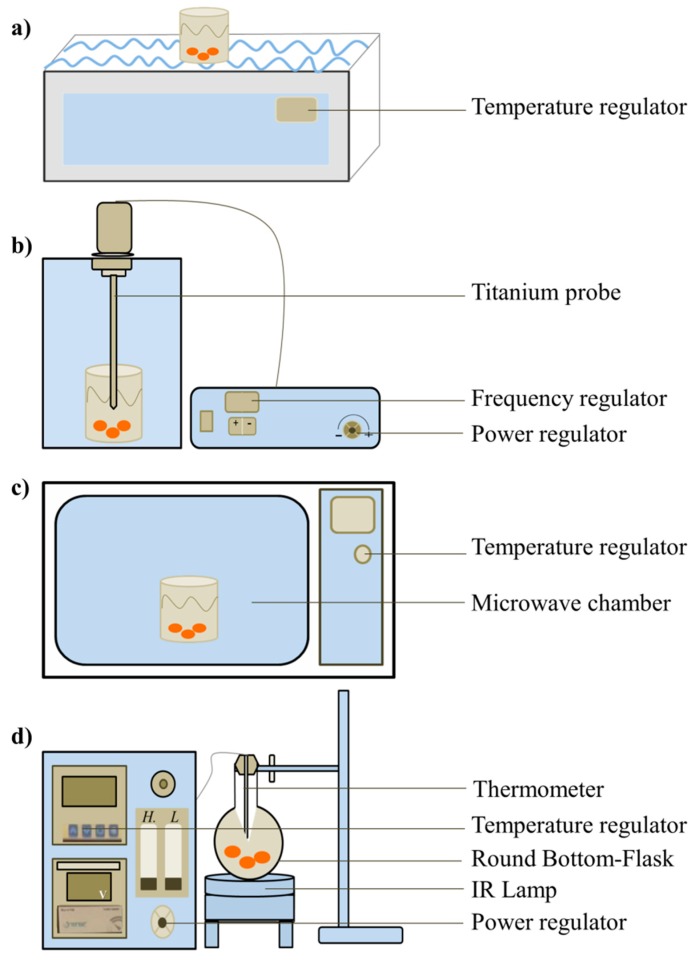
Instrumental set-up for the (**a**) solid-liquid, (**b**) ultrasound, (**c**) microwave, and (**d**) infrared methods.

The infrared-assisted extraction set-up was designed by the Faculté des Sciences, Université Saint Joseph de Beyrouth (Figure 1d). The extraction prototype consists of a ceramic infrared transmitter (power ranging from 63 and 170 W) for the heating process, linked to a proportional-integral-derivative (PID) control, where the temperature and voltage are adjusted automatically or manually. This novel method uses infrared energy emitted from the ceramic transmitter to heat the solvent matrix mixture contained in a round bottom flask and placed at a changeable distance away from the ceramic transmitter. To a round-bottom flask, 100 mL of preheated water (75 °C) and 10 g of apricot pomace was added. The flask was then fixed on the top of the ceramic transmitter. The temperature was set at 75 °C for 60 min (based on a preliminary study). Please note that some of the apricot pomace will dissolve, but most will be insoluble in water. The specific IR energy $W_{IR}\;\left(\mathrm{kJ}/\mathrm{kg}\right)$ was calculated using:

(1) $$W_{IR}=\frac{P_{IR}\times t_{IR}}{m}$$

where *P* is the generator power (70 J/s), *t* is the total treatment duration (s), *m* is the product mass (kg), noting that *m* = 110 g (100 g water + 10 g of apricot pomace), and *W* is the energy consumed (2290.90 kJ/kg).

#### 2.2.2. Solid-Liquid Extraction 

The solid-liquid extraction process was conducted by using a water bath (power of 660 W) (Figure 1a). 100 mL of preheated water (75 °C) was mixed with 10 g of apricot pomace in a glass flask. The temperature in the water bath was set at 75 °C. 

The time needed for the solid-liquid extraction, $t_{S/L}\;\left(s\right)$, to reach the energy required (2290.90 kJ/kg) was calculated as follows:

(2) $$t_{S/L}=\frac{W_{S/L\;}\times m}{P_{S/L}}$$

where *W* is the energy consumed (2290.90 kJ/kg), *P* is the generator power (660 J/s), *m* is the product mass (kg), and *t* is the total treatment duration (s).

#### 2.2.3. Ultrasound Extraction

The ultrasound apparatus (BANDELIN SONOPLUS, HD 2200, Berlin, Germany) (Figure 1b) was composed of an ultrasonic generator (maximum power of 200 W and a maximum frequency of 24 kHz) coupled to a titanium probe placed in a chamber. The probe was immersed in a glass flask containing 10 g of apricot pomace mixed with 100 mL of preheated water (75 °C). To avoid heating during the US extraction, the temperature of the mixture was controlled by placing the flask in a cold water bath.

The US energy was calculated as follows:

(3) $$W_{US}=\frac{P_{US}\times t_{US}}{m}$$

where *W* is the energy consumed (2290.90 kJ/kg), *P* is the generator power (200 J/s), *m* is the product mass (kg), and *t* is the total treatment duration (s).

#### 2.2.4. Microwave Extraction

The microwave used in this experiment is a traditional one (Kenwood, MW 572, Kanagawa, Japan) (Figure 1c) with a power of 900 W. The mixture containing 10 g of apricot pomace mixed in 100 mL of preheated water at 75 °C was placed inside the microwave chamber.

The MW energy was calculated as follows:

(4) $$W_{MW}=\frac{P_{MW}\times t_{MW}}{m}$$

where *W* is the energy consumed (2290.90 kJ/kg), *P* is the microwave power (900 J/s), *m* is the product mass (kg), and *t* is the total treatment duration (s).

### 2.3. Analysis

#### 2.3.1. Dry Matter Content

For the calculation of the dry matter content of the raw material, an appropriate amount (10 g) of sample was weighed and then dried for 24 h in a ventilated oven at 105 °C [9].

#### 2.3.2. Quantification of Total Polyphenol Content by the Folin-Ciocalteu Method

The quantification of the total polyphenols was done by mixing 0.2 mL of the apricot pomace extract, 0.1 mL of FC reagent, and 0.8 mL of sodium carbonate Na_2_CO_3_ (75 mg/L), and was then incubated at 60 °C for 10 min. The color generated was measured at 750 nm using a spectrophotometer UV-VIS (Gold S54T UV-VIS, Shanghai, China). The polyphenol concentration of the samples was evaluated compared to a calibration curve of gallic acid and determined as milligrams of gallic acid equivalent (GAE) per gram of dry matter of pomace (DM) (mg GAE/g DM) [10].

#### 2.3.3. Determination of the Antiradical Activity 

The scavenging activity of DPPH free radical was examined by the DPPH (1,1-diphenyl-2-picrylhydrazyl) scavenging method. 4 mL of 0.1 mM DPPH (dissolve in methanol) were mixed with 0.2 mL of the extracts and then kept at room temperature for 30 min. The reduction of the DPPH free radical was examined by reading the absorbance at 517 nm [11]. Methanol was used as a blank. Two models were represented; the percentage of inhibition at the initial concentration of the extracts and the inhibition percentage at a fixed polyphenol concentration (4 mg GAE/g DM) in order to test the influence of their diversity. The inhibition percentage of the DPPH free radical was calculated as follows:

(5) $$\%\;inhibition=\left[\frac{\left(absorbance\;of\;control-absorbance\;of\;test\;sample\right)}{absorbance\;of\;control}\right]\times 100$$

#### 2.3.4. Determination of Tannin Concentration

The determination of tannin content was determined to the method reported by Ribéreau-Gayon et al. Two tubes were prepared, each one containing 1 mL of the extract, 0.5 mL of water, and 1.5 mL of hydrogen chloride HCl (12 N). The first tube was heated at 100 °C for 30 min and the second one was kept at room temperature for the same duration. Then, 0.25 mL of ethanol was added after the rapid cooling of each tube and the absorbance was measured at 520 nm [12]. The tannin concentration was calculated as follows:

(6) $$Tannin\;concentration\;\left(\frac{mg}{L}\right)=19.33\times \Delta \;optical\;densities$$

#### 2.3.5. Determination of Total Flavonoids (TF)

The total flavonoids content was analyzed by mixing 1 mL of apricot sample extract and 4 mL of water. After 5 min, 0.3 mL of sodium nitrite NaNO_2_ (5%) and 1.5 mL of aluminium chloride AlCl_3_ (2%) were added. 2 mL of sodium hydroxide NaOH (1 M) was then added to the mixture solution after a brief incubation at room temperature for 5 min. The color generated was read at 510 nm. Total flavonoids content in samples was analyzed using the calibration curve of catechin and expressed as the mg of catechin equivalent (CE) per g of dry matter [13].

#### 2.3.6. HPLC-DAD Analysis

The polyphenol analyses of the extracts prepared from the apricot pomace were determined using high-performance liquid chromatography (HPLC). The apparatus consisted of a liquid-chromatography-Agilent 1100 Series system (Teknokroma Professional Friendly Lichrospher 100 RP18 5 µM, 25 × 0.46, Serial number NF-21378, Barcelona, Spain), an autosampler, a column oven, and a diode array detector. The separation and identification of the polyphenolic molecules was done using a C18 Column Zorbax (Barcelona, Spain) (25 × 0.46 mm). The volume injected was 10 µL and the flow rate was of 1 mL/min. The chromatographic conditions consisted of a mobile phase made up of acidified nanopure water at pH 2.3 with HCl (A) and acetonitrile (B). The elution was performed using isocratic conditions as follows: From 0 to 5 min with (85%) A and (15%) B. Gradient profile ranged from 5 to 30 min, beginning with (85%) A and (15%) B and ending with (0%) A and (100%) B. Isocratic conditions from 30 to 35 min with (0%) A and (100%) B were then done. Each compound was identified by comparing different retention times of each peak to those of the standards ones [14]. The quantification of the phenolic molecules was evaluated using the standard curve determined for each molecule by injecting various concentration of each standard: Epicatechin, catechin, and rutin. 

#### 2.3.7. Bacterial Strains, Culture Media, and Growth Conditions

The antibacterial activity was screened against eight different bacterial strains: Two strains of Staphylococcus epidermidis, amongst which, one is Methicillin resistant (MRSE 2380); two gram positive bacterial strains of Staphylococcus aureus, amongst which, one is methicillin-resistant staphylococcus aureus (MRSA 2); two gram negative strains of Klebsiella pneumonia, amongst which, one is Metallo-beta-lactamase producer; and two strains of Escherichia coli strains amongst which, one is extended spectrum beta lactamase (ESBL 2238).

#### 2.3.8. Preparation of the Bacterial Inocula for Minimal Inhibitory Concentration (MIC)

The bacterial strains were cultured on a freshly-prepared blood agar. Part of the colonies of each bacterial strain was inoculated in 3 mL of cation adjusted Mueller Hinton broth until reaching a turbidity of 0.5 McFarland. Then, a dilution of 1/100 was made into tubes of adjusted Mueller Hinton broth [15].

#### 2.3.9. Phenolic Extracts Preparation for MIC Assessment

The minimal inhibitory concentration assessment was done for each infrared, microwave, ultrasound, and solid-liquid extracts using five serial dilutions (starting from 2.5 to 40 µg/mL). Using 0.4 µm disposable syringe filters, all stocks were sterilized. The concentrations of the four different polyphenolic extracts were diminished to: 20 µg/mL and up to 1.25 µg/mL after adding equal volumes (100 µL) of the four apricot extracts to the different bacterial strains (100 µL) using 96 well (U shape) microtiter plates. After an overnight incubation at 37 °C, the microtiter plates of each polyphenolic extract were read in order to determine the MIC that blocks the bacterial growth.

### 2.4. Scanning Electron Microscopy (SEM)

The microstructure of the apricot pomace cells was investigated after extraction. Cells were first lyophilized at −55 °C (Ilshin Lab. Co. Ltd., Yangju-si, Korea). A conductive double layer carbon support coating and then gold coating (Quorum 150T ES, London, UK) was applied to the cells. Samples were then examined by scanning electron microscopy (SEM) (TESCAN MIRA3 FEG-SEM, Prague, Czech Republic) with a high vacuum voltage.

### 2.5. Statistical Analysis

Each experiment was repeated twice. The standard deviations and means were calculated. The standard errors correspond to the error bars in all figures. The Least Significant Difference test (LSD) as well as variance analyses (ANOVA) were calculated in order to determine the significant differences. The statistical analyses were carried out using STATGRAPHICS^®^ Centurion XV software (StatPoint Technologies, Inc., Warrenton, VA, USA).

## 3. Results and Discussion

### 3.1. Effect of Different Extraction Techniques on Polyphenols, Tannins, and Flavonoids Concentrations in Apricot Pomace Extracts

**Figure 2:**
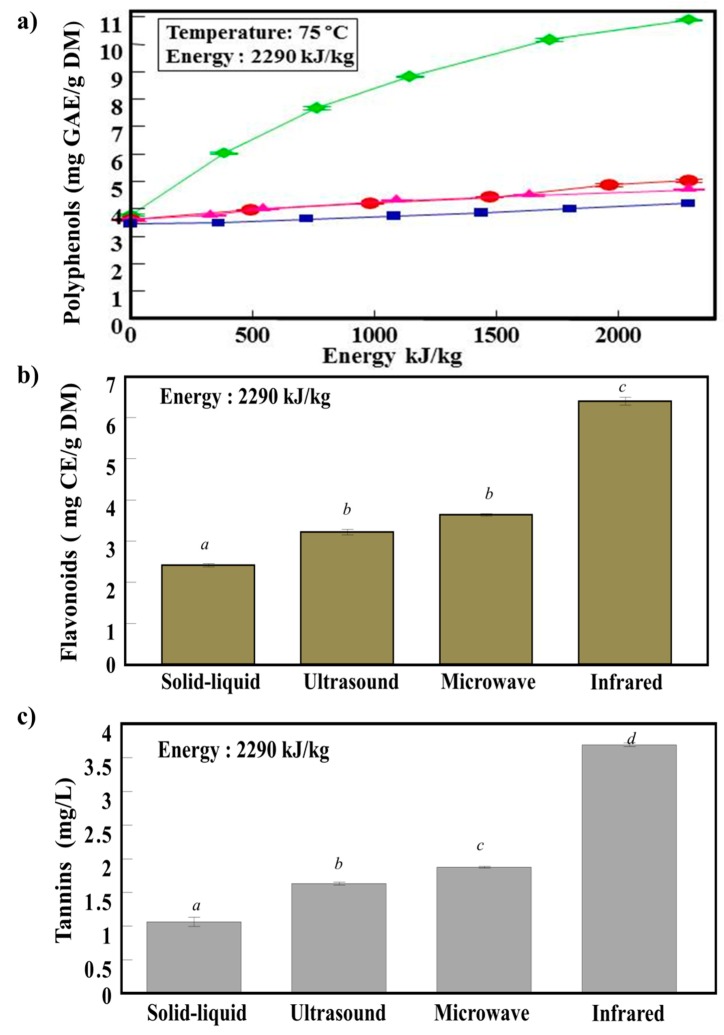
(**a**) Polyphenol yield of apricot pomace treated with 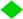 infrared, 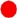 microwave, 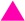 ultrasound, and 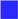 solid-liquid as a function of energy input. (**b**) Flavonoid and (**c**) tannin recovery for solid-liquid, ultrasound, microwave, and infrared of apricot pomace extracts. Different superscript letters indicate a significant statistical difference (*p* < 0.05).

Figure 2a presents the yield of polyphenols from apricot pomace, treated by different techniques (infrared, microwave, ultrasound, and solid-liquid extraction), as a function of the energy input. At the same energy (2290 kJ/kg), the maximal polyphenol yield was obtained by infrared (IR) (10.8 mg GAE/g DM) followed by microwave (MW) (5 mg GAE/g DM), ultrasound (US) (4.8 mg GAE/g DM), and solid-liquid (S/L), which exhibited the lowest polyphenol yield (4 mg GAE/g DM). At the same treatment duration (e.g., 5 min), IR also gave the highest polyphenol yield (5.3 mg GAE/g DM) compared to MW (5 mg GAE/g DM), US (4.3 mg GAE/g DM), and S/L (3.8 mg GAE/g DM). The efficiency of IR extraction can be attributed to the electromagnetic waves that excite the sample molecules in the modes of twisting, stretching, and bending [8,16]. In concordance with our results, Cai et al. (2011) showed that IR exhibited a higher extraction yield of procyanidin B2, catechin, and epicatechin from grape seeds compared to microwave-assisted extraction and ultrasounds [16]. The enhancement recovery using microwaves was attributed to its heating effect due to the rotation movement of the solvent inside the microwave chamber. This causes a rapid elevation of the solvent temperature, which increases the solubility of phenolic molecules [17]. Flavonoid and tannin recoveries for IR, MW, US, and S/L apricot pomace are presented in Figure 2b,c, respectively. In concordance with polyphenol content; IR was 1.5 and 2 times more efficient than US and MW in extracting flavonoids and tannins, respectively.

Each technique plays an important role in the extraction process from the sample matrix. IR radiation directly heats the sample matrix without heating the surrounding air, which could increase in the extraction of bioactive molecules. However, in conventional methods, a longer time is needed before the energy of heat is conducted and transferred to the sample matrix [16]. The effectiveness of the IR extraction method could be due to the matching between the absorption characteristics of the solvent used and the wavelength of the infrared heater [8].

### 3.2. Scanning Electron Microscopy of the Apricot Pomace Extracted by Different Techniques

**Figure 3:**
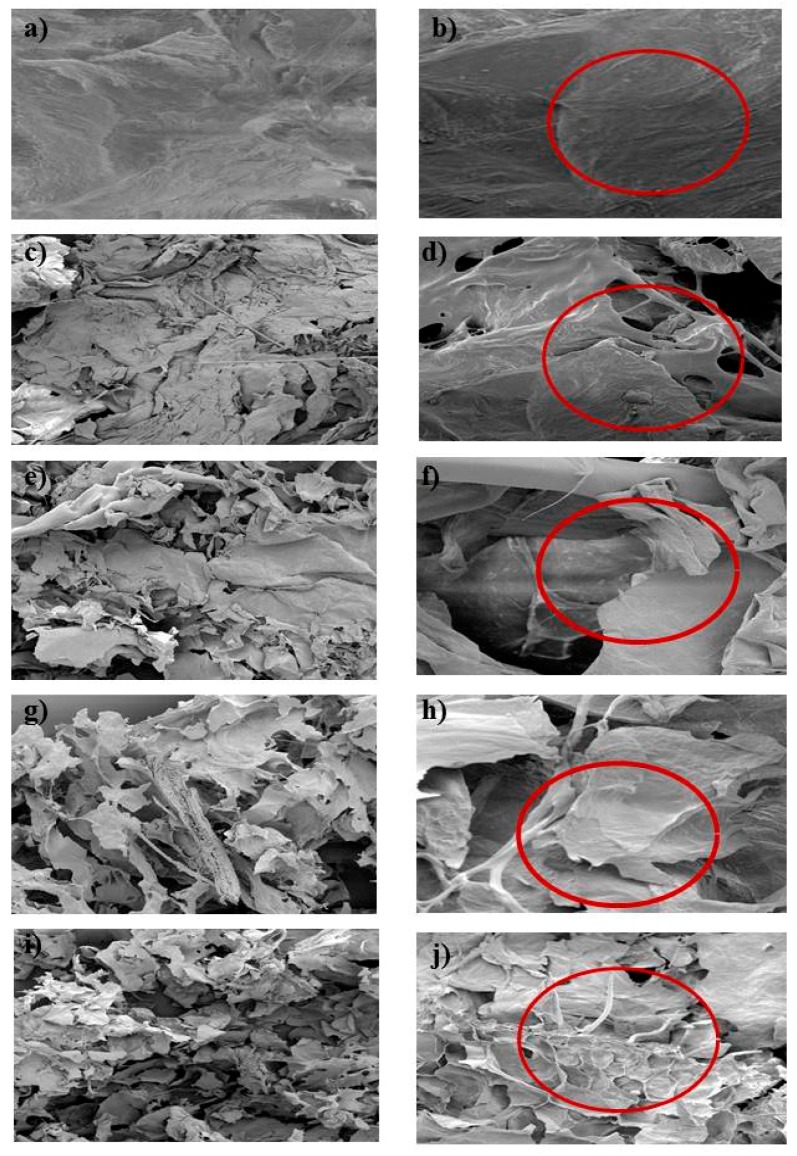
Scanning Electron Microscopy (SEM) of apricot pomace by 70× (**a**,**c**,**e**,**g**,**i**) and by 575× (**b**,**d**,**f**,**h**,**j**); (**a**,**b**), untreated, (**c**,**d**) solid-liquid, (**e**,**f**) ultrasound, (**g**,**h**) microwave, and (**i**,**j**) infrared.

The images of apricot pomace obtained by scanning electron microscope (SEM) prior to and after different treatments are shown in Figure 3. Compared to Figure 3a,b, which represented the intact cells of apricot pomace, solid/liquid (Figure 3c,d) showed partially damaged cells, followed by ultrasounds (Figure 3e,f) and microwaves (Figure 3g,h), which exhibited more ruptured cells. Infrared extraction (Figure 3i,j) showed the highest disrupted apricot pomace cells with a small presence of intact cells (shown in Figure 3j). The IR wave heating mechanism with its high efficiency was shown to result in high cell bursting [16]. The MW technique causes the rupture of cells because they are subjected to a localized pressure and thermal stress [18]. Concerning the US technique, the local pressure of the implosion air bubbles contributes to the damage of the proximate cellular membranes [19]. The SEM results are in concordance with the results obtained for polyphenol, flavonoid, and tannin, and indicate that the more ruptured and damaged the cells are, the more polyphenols are extracted.

### 3.3. Antiradical Activity of the Apricot Pomace Extracted by Different Techniques

**Figure 4:**
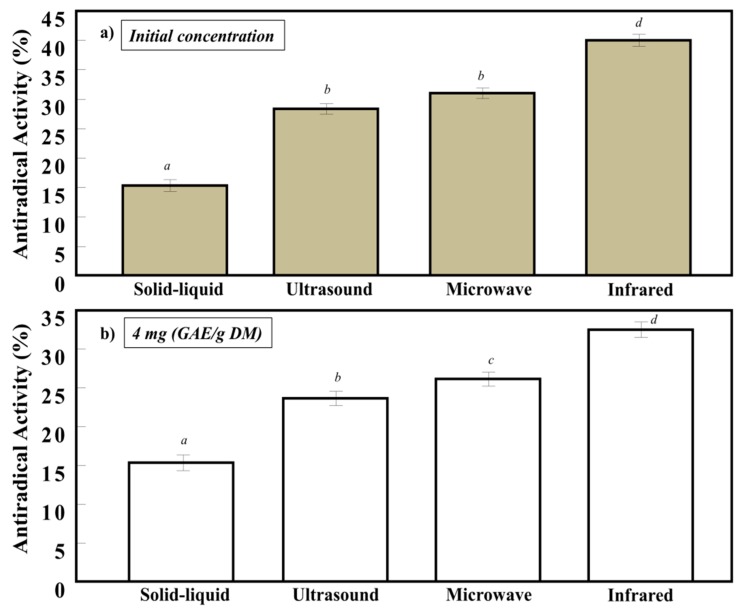
Antiradical scavenging capacity of apricot pomace treated with solid-liquid, ultrasound, microwave, and infrared extracts (**a**) at their initial concentration and (**b**) at 4 mg GAE/g DM of polyphenols. Different superscript letters indicate a significant statistical difference (*p* < 0.05).

Figure 4a shows the inhibition percentage obtained for the four extracts treated by different extraction techniques at their initial polyphenol concentrations. The higher the polyphenols, flavonoids, and tannins contents, the better the antiradical activity (AA) was. This was in concordance with many studies that showed the concentration-dependent antiradical activity of polyphenol extracts [20]. IR extract gave the highest inhibition percentage (40%) followed by MW (32%) and US (28%), and the lowest AA was obtained for the solid-liquid extraction (15%). On the other hand, and at the same polyphenol concentration (Figure 4b), IR exhibited the best AC than the other extracts. These results could be attributed to the different polyphenol composition, responsible for the enhancement of the radical scavenging activity [21].

### 3.4. Antimicrobial Activity of the Apricot Pomace Extracted by Different Techniques

The antimicrobial activities of the four apricot pomace extracts treated by IR, MW, US, and S/L techniques were tested against different gram-positive strains and gram-negative bacterial strains (Table 1) using MIC for the different concentrations of 20 µg/mL, 10 µg/mL, 5 µg/mL, 2.5 µg/mL, and 1.25 µg/mL. As it was expected, IR extracts of apricot pomace exhibited the lowest inhibitory concentration, denoting the highest inhibitory activity against gram-positive strains and one gram-negative strain. IR extract had also the highest flavonoid and tannin concentrations (Figure 2b,c), which play an important role in the bioactivity of polyphenols. The antimicrobial results are in concordance with the radical scavenging activity, where the IR extract gave the highest activity (Figure 4a,b). MW and US gave the same results and were active against all the gram-positive bacterial strains (whether they are *Methicillin resistant* or not) as well as against the gram-negative bacteria (*Escherichia coli*). However, their required inhibition concentration was more than that of IR. On the other hand, the S/L extract showed the lowest inhibition against two gram-positive coagulase negative bacteria since its phenolic content was the smaller compared to the other techniques. These results are in concordance with the findings of Naz et al. (2006), which showed that phenolic molecules have a strong activity against gram-positive strains [22]. The results of Table 1 exhibited that the effectiveness of polyphenols was higher against gram-positive bacterial strains compared to gram-negative ones. Those findings could be due to the fact that polyphenolic compounds have higher activity against gram-positive bacterial strains compared to gram-negative ones, since the latter possess an outer membrane in their cell wall acting as a barrier and consequently decreasing the uptake of these molecules. Moreover, a mutation in porin protein or the efflux phenomena in bacteria could also reduce the activity of polyphenol molecules against them [23,24].

### 3.5. Quantification of Polyphenol Extracts by High-Performance Liquid Chromatography

The composition of polyphenol in apricot pomace extracted by the different techniques was shown in Table 2. The main polyphenols in the extracts were rutin, catechin, and epicatechin (shown also in the supplementary Figure S1). These findings were consistent with the results of Veberic and Stampar (2005) on the polyphenol composition in apricot varieties [25]. Rutin was found in all extracts. However, catechin was detected in IR (3.1 μg/g DM), MW (2.1 μg/g DM) and US (1.5 μg/g DM). Epicatechin was only detected in infrared extract (4 μg/g DM). Our results are in concordance with the study of Cai et al. (2011), which showed that infrared technique gave higher extraction yields of catechin and epicatechin from grape seeds compared to microwave and ultrasound techniques [16]. The diversity of polyphenol could be related to the structure of the extracted molecules as well as the extraction conditions of each method [16]. IR showed the highest extraction yield for all the detected polyphenol compounds (Table 2). Epicatechin, catechin, and rutin were proven to be strong antioxidant molecules and have a synergistic effect, which contributes to high biological activities [26,27]. This polyphenol diversity could explain the highest biological activity (antiradical and antimicrobial activities) of infrared extract.

## 4. Conclusions

The comparison of different techniques on the extraction of polyphenols from apricot pomace showed that infrared was the most effective method, which gave the highest polyphenol, flavonoid, and tannin concentrations, followed by microwave, ultrasound, and solid-liquid extractions. Infrared has been shown to give the highest antimicrobial and antiradical activity. The scanning electron microscopy revealed that the IR technique induced the highest cellular and structural damage in apricot pomace, which could explain the effectiveness of this novel technique in extracting polyphenols. IR could be considered as a promising technology for food waste recovery.

## Figures and Tables

**Table 1:** Minimum inhibitory concentration (µg/mL) of different gram-positive and gram-negative bacteria obtained with different extraction techniques. (-) indicates the absence of inhibitory effects.

Bacteria/POMs	Minimum Inhibitory Concentration (µg/mL)			
	Infrared µg/mL	Microwave µg/mL	Ultrasound µg/mL	Solid-Liquid µg/mL
Methicillin-resistant *Staphylococcus aureus* (MRSA 2) (gram +)	10	20	20	-
*Staphylococcus aureus* 2030 (gram +)	10	20	20	-
*Methicillin-resistant Staphylococcus epidermidis* MRSE 2380 (gram +)	10	20	20	20
*Staphylococcus epidermidis* 2047 (gram +)	10	10	10	20
*Klebsiella* 118 metallo beta lactamse (gram −)	-	-	-	-
*Klebsiella* (gram −)	-	-	-	-
*Escherichia coli* ESBL 2238 (gram −)	-	-	-	-
*Escherichia coli* (gram −)	10	20	20	-

**Table 2:** Polyphenols’ composition by High Performance Liquid Chromatography obtained with different extraction techniques. Different superscript letters indicate a significant statistical difference (*p* < 0.05). Nd: Non-detectable.

Techniques	Rutin (μg/g DM)	Catechin (μg/g DM)	Epicatechin (μg/g DM)
Solid-liquid	1.6 ^a^	Nd	Nd
Ultrasound	2.1 ^b^	1.5 ^c^	Nd
Microwave	1.7 ^c^	2.1 ^b^	Nd
Infrared	2.6 ^d^	3.1 ^d^	4 ^f^

## Supplementary Materials

The following are available online at http://www.mdpi.com/2076-3921/7/12/174/s1, Figure S1: Chromatographic profile of the several phenolic compounds.
