# Supplementary material for: Study of the Selectivity and Bioactivity of Polyphenols Using Infrared Assisted Extraction from Apricot Pomace Compared to Conventional Methods
Source: Antioxidants (Basel). 2018 Nov 27;7(12):174. doi: 10.3390/antiox7120174 (PMC6315536; doi:10.3390/antiox7120174)
Supplement: Supplementary file 1 [file antioxidants-07-00174-s001.pdf]

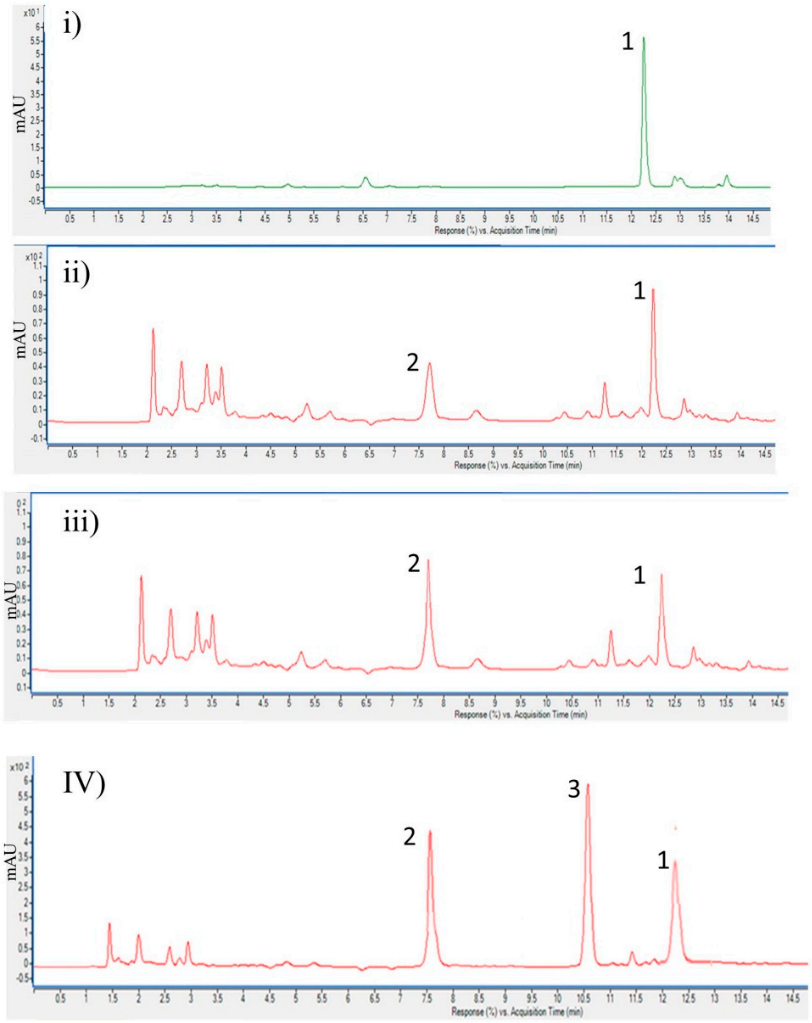

Figure S1. Chromatographic profile of the several phenolic compounds. 1: rutin, 2: catechin and 3: epicatechin of: i: solid-liquid, ii: ultrasound, iii: microwave and iv: infrared.
